# How pragmatic are randomised controlled trials evaluating minimally invasive surgery for oesophageal cancer? A methodological review of trial design using the Pragmatic-Explanatory Continuum Indicator Summary-2 (PRECIS-2) tool

**DOI:** 10.1136/bmjopen-2023-078417

**Published:** 2024-12-20

**Authors:** Katy Chalmers, Sian Cousins, Natalie S Blencowe, Jane Blazeby

**Affiliations:** 1 Centre for Surgical Research, University of Bristol, Bristol, UK

**Keywords:** Thoracic surgery, Clinical Trial, Oesophageal disease, Systematic Review

## Abstract

**Abstract:**

**Background:**

Surgical interventions are inherently complex and designing and conducting surgical randomised controlled trials (RCTs) can be challenging. Trial design impacts the applicability of trial results to clinical practice. Given the recent growth in numbers of surgical RCTs, there is a need to better understand the validity and applicability of trials in this field.

**Objectives:**

To examine the applicability and validity of RCTs comparing minimally invasive and open surgery for oesophageal cancer and to delineate areas for future research.

**Eligibility criteria:**

RCTs comparing open with minimal invasive oesophagectomy, published January 2012–June 2023. Abstracts, pilot and feasibility studies, and systematic reviews were excluded.

**Sources of evidence:**

Three sequential searches of Ovid MEDLINE, Embase and CENTRAL electronic databases and clinical trials registry databases.

**Charting methods:**

Two independent reviewers screened the articles and used appropriate, validated tools (Pragmatic-Explanatory Continuum Indicator Summary-2 (PRECIS-2) and Risk of Bias 2) to assess study quality. Trials were considered pragmatic if they were conducted in multiple centres and had a mean score of four or above on the PRECIS-2.

**Results:**

Nine RCTs were identified. One was judged to be pragmatic. The remaining eight were limited by narrow eligibility criteria, being single-centred or having strict intervention protocols. Two studies were low risk of bias, of which one was pragmatic, and three high, due to unblinded outcome assessment. The remaining four studies were of ‘some concern’ due to poor reporting.

**Conclusions:**

Only one trial identified in this review was considered pragmatic. More lenient criteria, as used in other reviews, may increase the proportion. There is a need for clearer guidance on the cut-off values that define a trial as pragmatic. It is recommended that the intended purpose of the trial, whether explanatory or pragmatic, receives more attention during surgical trial study design and conduct.

**STRENGTHS AND LIMITATIONS OF THIS STUDY**

- This review provides a comprehensive assessment of applicability and validity by two reviewers independently, using validated tools.
- Due to the original purpose of Pragmatic-Explanatory Continuum Indicator Summary-2 (PRECIS-2), there is no recognised standardised method for using PRECIS-2 scores to define trials as explanatory or pragmatic.
- We provide detailed text to ensure transparency about how the PRECIS-2 and Risk of Bias 2 tools were applied.

## Introduction

 Surgical interventions are inherently complex. They involve multiple components and co-interventions,^1^,^5^ delivered by collaborating healthcare professionals with varying expertise, in complex environments. These factors require consideration during the design and conduct of randomised controlled trials (RCTs) as they determine how applicable trial results are to usual practice and how they may be used to inform decision-making.

Validity, which refers to the extent to which the observed treatment effect may be due to biases,^6^ is widely understood and accepted. Pragmaticism (ie, how applicable trial results are to usual care) is a less well recognised concept. A pragmatic trial aims to reflect ‘real-world’ settings to inform decisions to adopt interventions into clinical practice.^7^ For example, these trials would have multiple experienced surgeons delivering interventions, in numerous centres across a wide range of eligible patients. In contrast, an explanatory trial is conducted in a tightly controlled, ‘idealised’ setting,^7^ for example, involving one or two expert surgeons, in a single centre, with a narrow subset of eligible patients.^8^ When assessing evidence from RCTs, it is essential to consider both the validity of the trial and how applicable (pragmatic) the trial is.

Given the recent growth in numbers of surgical RCTs, there is a need to better understand the validity and applicability of trials in this field. The aim of this study was to examine RCTs comparing minimally invasive and open surgery for oesophageal cancer, focusing on applicability to delineate areas for future research. This case study was chosen because it is complex with possible variation in eligibility criteria (eg, exclusion of patients with specific tumour gradings or co-morbidities), intervention delivery (eg, location of anastomoses and extent of lymph node dissection) and contextual variables that may impact intervention delivery and outcomes (eg, surgeon, team and centre experience and training, access to equipment such as robots). All these trial design characteristics can impact the applicability of results, and so it is crucial that it is considered. To our knowledge, none of the existing systematic reviews in this area have considered this issue.^9^,^16^

## Methods

Annual literature searches were undertaken to inform the steering committee of an ongoing RCT (Randomised Oesophagectomy: Minimally Invasive or Open (ROMIO))^17^ of up-to-date evidence. Eligible studies were assessed for applicability and validity using validated tools. Data were synthesised narratively, and key findings presented.

### Study eligibility

Included were RCTs (including ancillary studies, substudies and long-term follow-ups) comparing open with minimally invasive oesophagectomy in patients with oesophageal cancer. Any variant of minimally invasive oesophagectomy (total, hybrid or robotic) was eligible. Protocols of included RCTs and clinical trial registry database entries were retrieved where cited, and where protocols were not available, corresponding authors were contacted and asked to provide a copy. Reviews, conference abstracts, feasibility, pilot studies and those reporting only technical components of procedures were excluded.

### Searches and screening

Three annual consecutive searches and a final update search of electronic databases (Ovid MEDLINE, Ovid Embase and CENTRAL) were conducted for RCTs published (i) January 2012–December 2017, (ii) January 2018–November 2018, (iii) December 2018–November 2020 and (iv) December 2020–June 2023. Comprehensive search strategies (online supplemental table 1) were tailored for each database using the concepts oesophageal cancer, open surgery, minimally invasive surgery and RCTs. Retrieved articles were imported into Endnote (V.20) and deduplicated. One assessor (KAC) screened titles and abstracts. Full texts of remaining articles were retrieved and assessed for eligibility. If eligibility was unclear, a second reviewer (SEC) was approached, and if necessary, a third senior reviewer (JMB). Identified studies were presented and discussed with the ROMIO study management group, comprising experts in oesophageal cancer. This provided assurance that the searches and screening were comprehensive.

**Table 1:** Detailed characteristics of included studies

Study number	First author (publication year)	Countries	Number of centres	Number of patients randomised		Type of MIO	Primary outcome* (timing of outcome)
				Open	MIO		
1a	Biere (2012)	Netherlands/Spain/Italy	5	56	59	Total	Pulmonary infection (2 weeks)
1b†	Maas (2014)	Netherlands	1	13	14		Stress response measurements (1 day)
1c†	Maas (2015)	Netherlands/Spain/Italy	5	56	59		HRQL (1 year)
1d†	Biere (2017)			56	59		Factors associated with respiratory infections (not stated)
1e†	Straatman (2017)			56	59		Overall survival (3 years)
2	Guo (2013)	China	1	110	111	Total	Length of operation (not applicable)
3	Hong (2013)	China	1	59	55	Total	Pulmonary infection (2 weeks)
4	Paireder (2018)	Austria	1	13	16	Hybrid laparoscopy and thoracotomy	Morbidity and mortality (4 weeks)
5	Ma (2018)	China	1	97	47	Total	Morbidity (not stated)
6	van der Sluis (2019)	Netherlands	1	56	56	Totalrobot-assisted	Morbidity (2 weeks)
7a	Mariette (2019)	France	13	104	103	Hybrid laparoscopy and thoracotomy	Morbidity (4 weeks)
7b‡	Mariette (2019)			104	103		HRQL (3 years)
8	Zhang (2020)	China	1	50	50	Total	Length of operation (not applicable)
9	Yu (2022)	China	1	45	45	Total	Length of operation (not applicable)

* If primary outcome was not specified, the first outcome listed in the abstract is given. For ancillary studies and substudies, this will differ from the original trial and will be the main outcome reported.

† Ancillary and sub-studies of Biere *et al.*^26^

‡ Publication of secondary outcomes.

HRQL, health-related quality of life; MIO, minimally invasive oesophagectomy

### Data extraction

Data extraction focused on three main aspects: key trial characteristics and assessments of pragmaticism and validity. Where multiple publications and/or protocols related to the same RCT, data were extracted on a per trial, rather than per paper, basis. Data were extracted and assessments made by two researchers (KAC, SEC); a third reviewer (JMB) was consulted for technical advice or if disagreements occurred. Key trial characteristics included the type of surgical intervention (eg, totally robotic oesophagectomy), number of participating centre and patients, and study countries.

### Assessment of pragmaticism

The Pragmatic-Explanatory Continuum Indicator Summary-2 (PRECIS-2) tool^7 18^ was used to assess characteristics of included RCTs that are important in determining applicability across nine domains (online supplemental table 2). Each domain was scored from 1 (very explanatory) to 5 (very pragmatic). Disagreements were discussed to facilitate determination of ‘rules’ to standardise judgments,^19^ in discussion with a third senior reviewer (JMB). Trials were then re-examined.

Aspects of PRECIS-2 that required specific attention because the interventions were surgical are explained here. ‘Flexibility: delivery’ addresses how much flexibility in the delivery of the intervention is allowed within the trial, for example, whether a strict prescribed protocol was used or whether delivery was at the surgeon’s discretion. Judgments of procedural standardisation were informed by deconstructing interventions using a validated typology^20 21^ in consultation with a senior surgeon with procedural knowledge of both interventions (JMB). ‘Flexibility: adherence’ looks at whether the intervention was delivered as intended and typically relates to a patient’s compliance with the intervention, such as taking medication. In surgical trials, patients are not involved in intervention delivery; PRECIS-2 authors therefore suggest this domain should be left blank.^7^ However, intervention adherence by surgeons may be examined by using methods to examine whether interventions were performed as planned (eg, by videorecording procedures). It is an important aspect of a surgical trial and merits individual acknowledgement.^22^ Therefore, for this review, ‘flexibility: adherence’ was assessed as more explanatory where trials had very detailed protocols requiring strict surgeon adherence and more pragmatic in trials where few components were specified as required (or prohibited). In both types, videotaping can be used to examine adherence to intervention delivery protocols.

PRECIS wheels^7^ were created to illustrate assessments for each domain, which were examined in conjunction with overall mean scores to provide a descriptive summary of assessments. In line with PRECIS-2 guidelines, individual domains scoring four or five were considered pragmatic; three, equally explanatory and pragmatic; and two or one, explanatory. As there is no recognised standardised method for using the scores across domains to define trials as explanatory or pragmatic, in the current review, trials were considered to be pragmatic if they were conducted in multiple centres^23^ and had a mean score of four or above. Descriptive accounts of all domains are provided for each trial.

### Assessment of validity

The Risk of Bias (ROB) 2 tool^24^ was used to assess validity. The judgments of five domains (online supplemental table 3) are considered collectively to produce an overall ROB (low risk, some concerns or high risk). Judgments were informed by the tool guidance^12^ and inputted into an Excel-based macro with in-built algorithms used to compute overall ROB for each trial. In the first instance, RCTs were assessed independently by two researchers (KAC and SEC). Assessments were then discussed to facilitate determination of ‘rules’ to standardise judgments, and papers re-assessed. Disagreements were resolved by discussion or by JMB. Verbatim text from papers was extracted and used as a narrative to support judgments.

### Assessment of inter-rater agreement

The extent of agreement between reviewers’ assessments of external and internal validity was determined by calculating the number of assessments that matched, expressed as a percentage.

### Patient and public involvement

Patients and public were not involved in the design or conduct of this systematic review.

### Ethics approval

Ethics approval was not required for this review as there were no participants involved.

## Results

**Figure 1:**
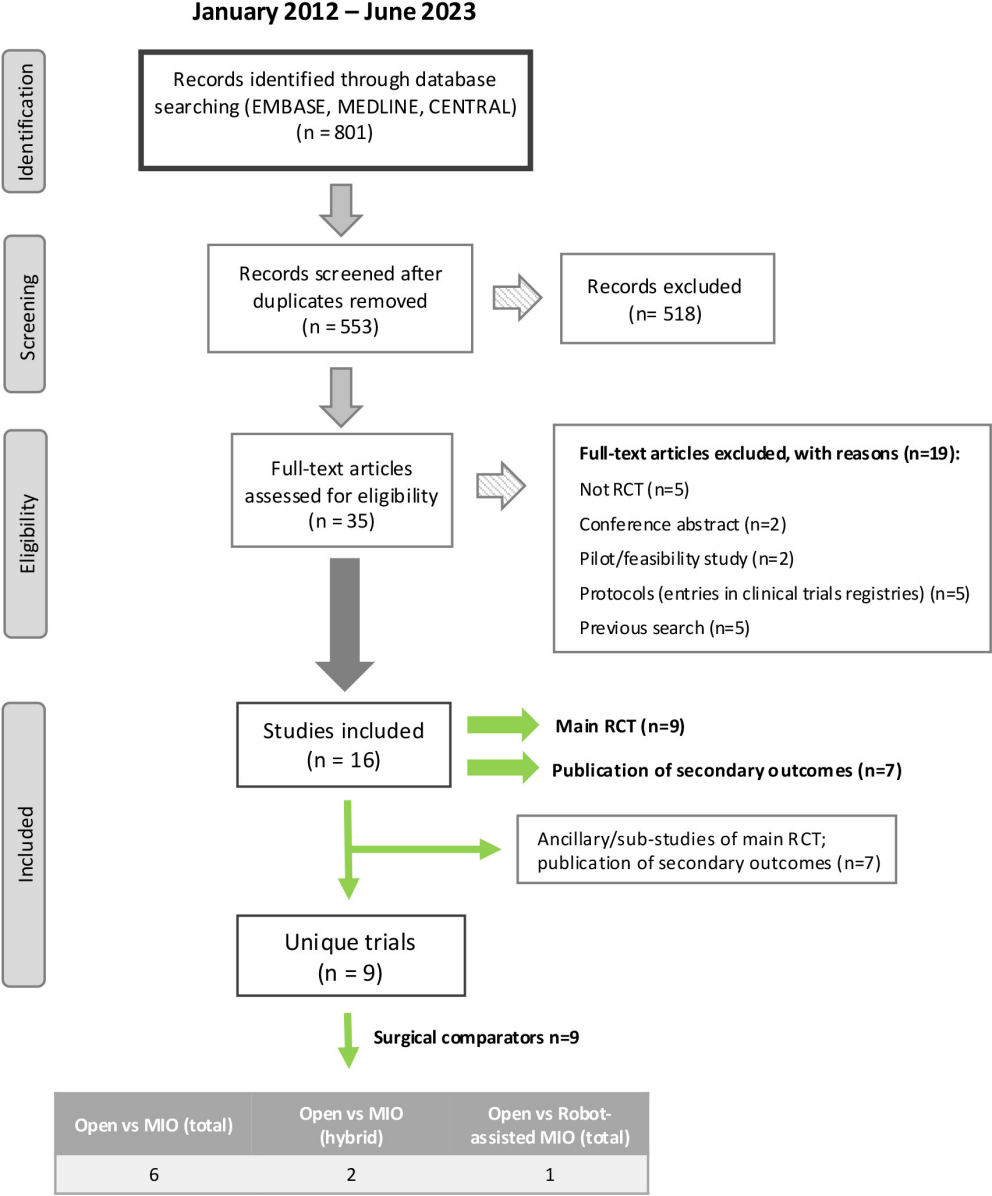
PRISMA flowchart depicting the search strategy and selection of articles for the review. MIO, minimally invasive oesophagectomy; PRISMA, Preferred Reporting Items for Systematic reviews and Meta-Analyses. RCT, randomised controlled trial.

Full texts of 35 articles were reviewed and nine RCTs included^25^,^38^ (figure 1).

### Key trial characteristics

Most RCTs (n=6) compared open and totally minimally invasive oesophagectomy (one robotically assisted) (table 1). Seven randomised <200 patients, including three with ≤100 patients. Seven were single centred. Five were conducted in China, one in each of the Netherlands, Austria and France and one was international (the Netherlands, Spain and Italy). Four studies^25 33 34 36^ calculated and reported sample sizes—one study^34^ was under powered as it did not recruit sufficient patients.

### Pragmaticism

**Figure 2:**
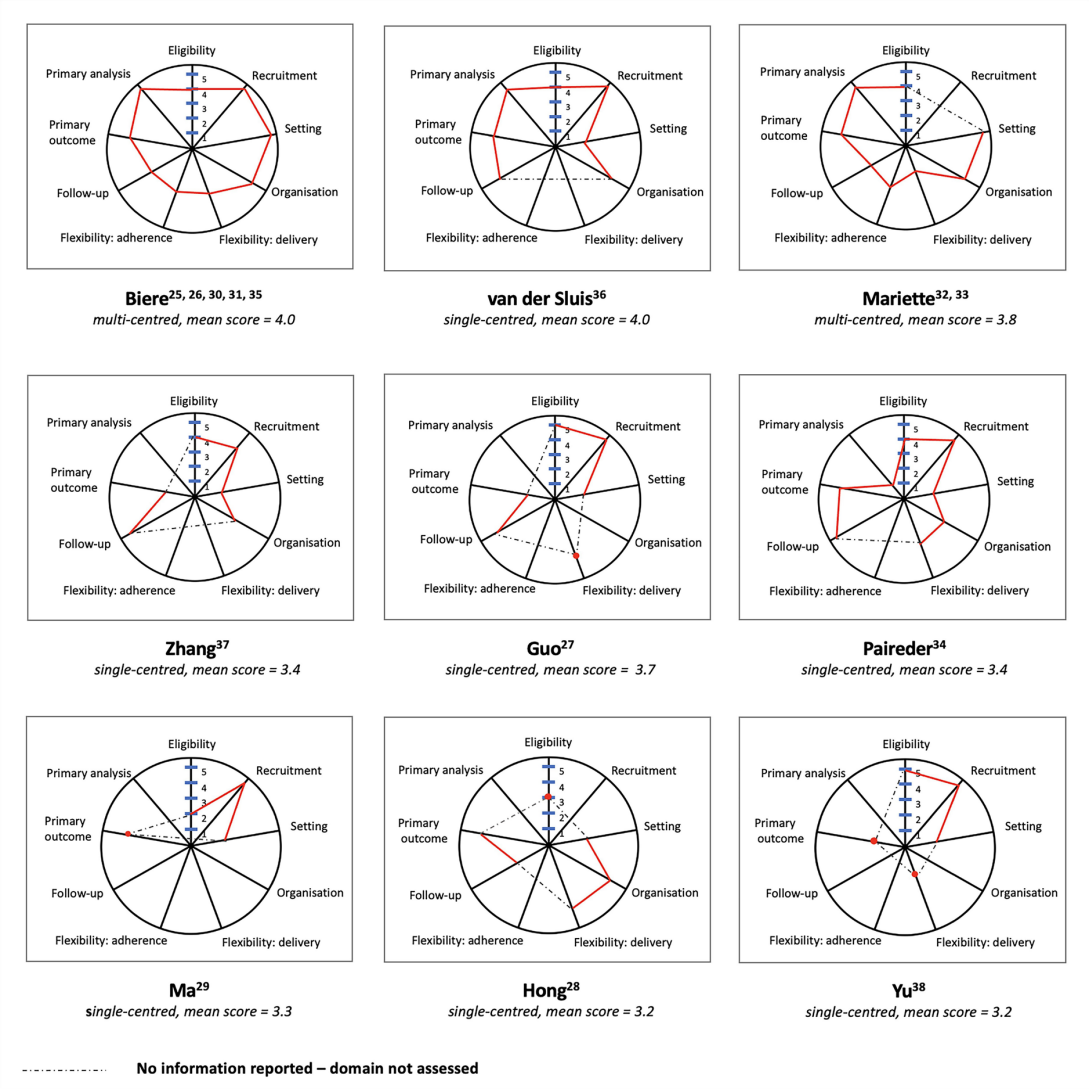
Pragmatic-Explanatory Continuum Indicator Summary-2 (PRECIS-2) scores for each domain plotted on PRECIS wheels, illustrating the pragmatism of included studies.

Only one study provided information to enable PRECIS assessments in all nine domains.^26^ No information was included for ‘flexibility: adherence’ in seven trials, ‘primary analyses’ in five trials and ‘organisation’ in four trials. Eligibility, setting and primary outcome were the only domains that could be populated for all (figure 2 and online supplemental table 4).

One study^25 26^ was considered to have adopted a more pragmatic approach as it was multicentred, recruited patients from outpatient clinics and performed an intention-to-treat analysis. However, additional data collection and standardisation and monitoring of interventions suggested that there were some aspects of trial design that were explanatory.

Most commonly, the recruitment domain was judged as pragmatic across studies.^2627 29 34 36^,^38^ ‘Primary analyses’ was reported by four of the nine included studies, three^26 33 36^ used intention-to-treat analysis, ensuring that patients were analysed within the group they were randomised, and one^34^ used per protocol analysis. The domains commonly judged to be more explanatory were ‘trial setting’ (most (n=7)^27^,^2934 36^ were conducted in single centres), ‘flexibility: delivery’ (four^26 33 34 38^ reported methods to standardise the delivery of interventions) and ‘flexibility: adherence’ (two^26 33^ reported strategies to monitor adherence to intervention delivery).

### Validity

**Figure 3:**
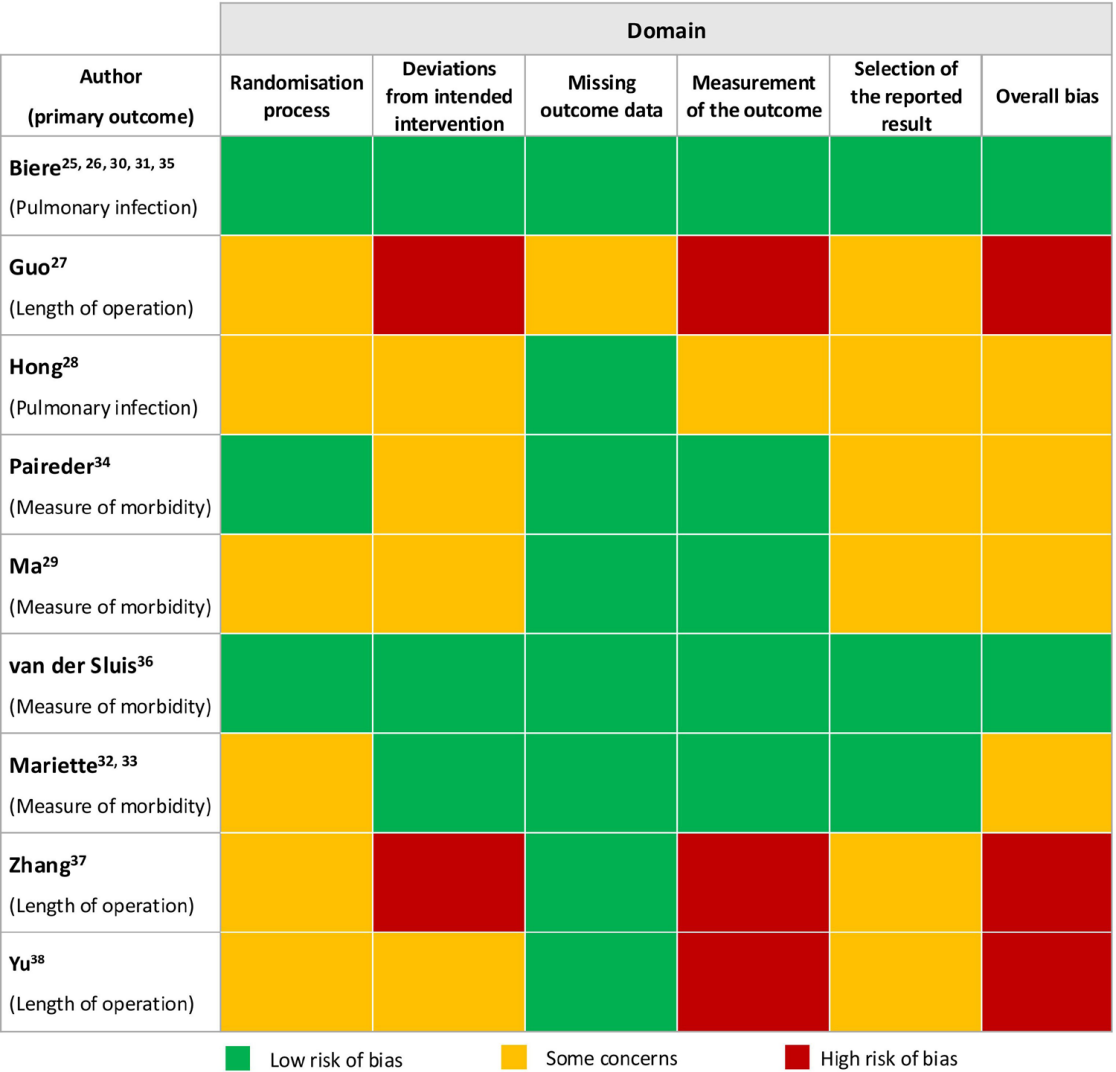
ROB assessment for the trial’s primary outcome, using the ROB 2 tool. ROB, risk of bias.

Overall ROB was judged to be low in two studies.^26 36^ Of the 45 individual judgments made over the five domains, 23 were low ROB. ‘Missing outcome data’ was most frequently judged to be at low ROB.^2628 29 33 34 36^,^38^ ‘Measurement of outcome’ was judged as low ROB in five studies; these used methods deemed to be appropriate to assess primary outcomes unlikely to be affected by lack of outcome assessor blinding.^27 28 37^ In six studies, there was insufficient information to make a judgment about ‘deviations from intended interventions’ as no information was reported about blinding of patients, carers or people delivering the intervention, crossovers and whether an intention-to-treat analysis was used. Only three trials^26 34 36^ comprehensively reported methods of randomisation and allocation (figure 3 and online supplemental table 5).

### Assessment of inter-rater agreement

After establishing rules to standardise judgments, agreement for PRECIS-2 assessments was 91.7% (mean, SD 9.8). For validity, agreement after initial judgments was 65% (mean, SD 20.7), with the greatest agreement for ‘missing outcome data’ (100%) and ‘randomisation process’ (75%) domains and least for the ‘deviations from intended interventions’ (37.5%) domain. Following discussions with a ROB expert (JS), consensus was consequently reached for all judgments across all domains after re-assessment.

## Discussion

This review provides an in-depth examination of surgical RCTs comparing two widely used procedures for oesophageal cancer (open and minimally invasive oesophagectomy) and whether outcomes from these trials are applicable to clinical practice. Until recently, applicability (also referred to as generalisability) has received much less attention than validity. Applicability was assessed using the PRECIS-2 tool.^7^ In the nine RCTs identified, only one reported information required to assess all nine PRECIS-2 domains. One study was classified as more pragmatic and therefore applicable to the wider clinical setting. The others had characteristics deemed more explanatory, including inclusion of only single centres, with narrow eligibility criteria, prescribed intervention delivery methods or an intense follow-up regime, characteristics not exhibited in ‘real-world’ clinical practice. There is a need for increased awareness of the importance of applicability within the surgical trials’ community if trial results are to be applied to a wider clinical setting. Higher quality pragmatic, multicentre surgical RCTs are required to generate evidence to inform clinical practice.

Defining trials as ‘explanatory’ or ‘pragmatic’ based on their intended purpose were first reported in 1967.^39^ A trial to determine the effects of interventions delivered in conditions more aligned with usual care was described as being pragmatic,^39^ and there may be more confidence that trial results are applicable to the wider target patient population. Certain trial characteristics are key in determining the degree of pragmatism. For example, study populations are determined by eligibility criteria; a pragmatic study would aim to include patients who would receive the intervention in usual care. Four of the RCTs examined excluded patients with tumours of the upper oesophagus,^26 33 34 36^ although these patients would have received the intervention in usual clinical practice.^33 34^ The number of study centres is also important; outcomes from one centre may not be comparable to another that differs in geography, culture or surgeon/centre experience. Only two studies were conducted in multiple centres.^26 33^ Dal-Ré *et al* suggested that single-centre studies should be considered ‘explanatory’ regardless of other domain scores,^23^ although they may be useful in decision-making where that the single centre is representative of the wider context.^18^ Treatment effects have been found to be larger in single centre studies,^40 41^ and so results should be interpreted with caution. It is only in multicentre trials that the intervention may be evaluated in different contexts with multiple surgeons.^37 38^ The importance of selecting centres that reflect those where procedures are delivered in usual care is highlighted by a comparison of data from the TIME trial^26^ (a multicentred European trial) with data from the Dutch Upper Gastrointestinal Cancer Audit, following the implementation of totally minimally invasive oesophagectomy in the Netherlands.^42^ Audit data showed increased total and pulmonary complications and reoperations in patients undergoing the minimally invasive intervention^42^—adverse events not seen in the TIME trial.^26^ It was concluded that this may reflect non-expert surgeons outside of high-volume centres performing this minimally invasive technique in a non-standardised fashion outside of a controlled trial environment.^42^ Assessment of applicability of the TIME trial using the PRECIS-2 tool in this review recognised that the experience required for both the centres and individual surgeons to complete the MIO was greater than for open oesophagectomy. However, Markar’s study^42^ has illustrated that perhaps a greater emphasis needs to be placed on the potential learning curve for surgeons and the experience of a non-specialist centre. This is a crucial point; for trial outcomes to be applicable, surgeons and centres conducting the interventions in RCTs need to reflect surgeons and centres nationally and perhaps internationally.

The majority of the RCTs examined were poorly reported. Tools such as the Standard Protocol Items: Recommendations for Interventional Trials checklist^43^ have been developed to encourage triallists to address (and report) specific items in interventional trials, including rationale, objectives, study setting, PICO (population, intervention, comparator, outcome), sample size and recruitment strategies. The provision of this information would enable a more informed, comprehensive assessment of a trial’s applicability. The Consolidated Standards of Reporting Trials checklist^44^ and subsequent extensions recommend that the generalisability of a trial’s findings should be discussed^45^ and suggest greater depth of reporting throughout all sections of a paper to facilitate judgements about applicability.^46^ Cited over 1100 times, the checklist is evidently recognised, yet poor reporting of key methodological aspects was evident in the studies examined. This is reflected in other reviews in surgery^47^ and medicine.^48^

Some trial design features, such as intervention standardisation and monitoring adherence, are judged to be explanatory by PRECIS-2; however, in surgical trials, their absence may compromise internal validity. For example, some standardisation of surgical procedures may be required, so interventions can be compared across surgeons/centres and to monitor their delivery. In terms of analysis, data from complex surgical trials should also be carefully considered when applying PRECIS-2. Strict intention-to-treat (ITT) analyses state that all patients should be analysed in their assigned group regardless of whether they receive an intervention, crossover or are lost to follow-up. Often in surgical trials, patients are found to no longer be eligible when they undergo surgery for reasons such as advanced disease or the presence of previously unknown pathologies, both which result in patients not receiving the allocated intervention. The undertaking of preplanned sensitivity analyses, such as modified ITT, may be seen as less pragmatic by PRECIS-2; however, in real terms, the provision of these analyses provides an important perspective about the results and their interpretation. The use of per-protocol analyses in surgical trials is therefore important and often essential in non-inferiority designs. The definitions of a per-protocol analyses in a surgical trial, however, are often confusing and trialists need to carefully describe this in relevant documents to optimise transparency. Comprehensive and transparent reporting will facilitate more informed considerations about applicability to be made.

Strengths of the review include rigorous and comprehensive assessment of applicability and validity by two reviewers independently, including sourcing unpublished protocols. Detailed text provides transparency about how the ROB-2 and PRECIS-2 tools were applied. Furthermore, this review addresses a gap in the literature in this area, and it modifies the PRECIS-2 methods to make them more applicable to surgical interventions by using a published typology. A potential limitation to the review is that due to the nature of the annual literature reviews, dual screening was not conducted. However, the narrow search criteria meant that the maximum number of entries to screen was <250, limiting this possibility, and findings were presented to experts involved in the recently completed ROMIO study^17^ to ensure comprehensiveness. A further point of note is that the PRECIS-2 tool was developed to aid triallists in the design stage rather than the assessment of completed studies. However, it is being used retrospectively,^23^ with some adjustments.^18^ This, and its ability to facilitate a comprehensive and detailed assessment of trial design features important in applicability, meant that the current review used the PRECIS-2 tool. However, it should be noted that there is no recognised standardised method for using PRECIS-2 scores to define trials as explanatory or pragmatic. Readers are encouraged to look at individual domain scores depicted in the PRECIS wheels, to gain a more in-depth understanding of each trial’s design.

Since completing this work, several publications have used,^49 50^ or propose to use,^51 52^ PRECIS-2 to determine the applicability of eligible studies in systematic reviews. Each has selected a different value to define a trial as pragmatic. Applying the criteria used by other authors to the eight trials identified in this review would have led to different conclusions. One method^50^ would have identified three studies^26 33 36^ as pragmatic, and the other three methods^49 51 52^ would have considered all studies as pragmatic.^26^ This is because a more lenient approach to study parameter classification^26^ was used in these reviews than in this review. It is recommended that standardised methods are established to interpret retrospective assessments to enable comparisons between studies. It is also recommended that new trials are clear about their intentions in design (ie, whether explanatory or more pragmatic) because of the lack of clarity seen in the included trials. This could be part of the trial protocol and accompanying publication. Until this is achieved, transparent reporting of all judgments and data, as reported in this review, are vital. In the area of oesophageal cancer surgery where robotic techniques are becoming more widespread, it is recommended that pragmatic trials are conducted maybe comparing robotic with minimal access techniques. A potential method to optimise investment would be to nest an IDEAL phase 2b more explanatory study within a larger pragmatic trial. The Ideal 2b study would only run in a few centres.^53^

Using RCTs of oesophagectomy for oesophageal cancer as a case study, this review has demonstrated that although trials contained both pragmatic and explanatory characteristics, only one was classified as pragmatic. Stakeholders involved in surgical trials need to be aware of the importance of applicability. Further work in this area should raise awareness of existing guidelines^43^,^4654^ and tools^7 24^ developed to improve the design, conduct and reporting of trials. It is also recommended that current methods are applied to reviews of RCTs of other surgical interventions to examine whether their findings are applicable. Triallists should be encouraged to incorporate considerations of applicability into the design stages of trials to ensure they are fit for the purpose. While well-designed, powered explanatory studies are possible and appropriate where the objective is to establish mechanisms and how it works in expert hands, A pragmatic approach is required in order to change clinical practice.

## supplementary material

10.1136/bmjopen-2023-078417 online supplemental file 1

## Data Availability

All data relevant to the study are included in the article or uploaded as supplementary information.
