# Supplementary material for: How pragmatic are randomised controlled trials evaluating minimally invasive surgery for oesophageal cancer? A methodological review of trial design using the Pragmatic-Explanatory Continuum Indicator Summary-2 (PRECIS-2) tool
Source: BMJ Open. 2024 Dec 20;14(12):e078417. doi: 10.1136/bmjopen-2023-078417 (PMC11664389; doi:10.1136/bmjopen-2023-078417)
Supplement: online supplemental file 1 [file bmjopen-14-12-s001.pdf]

Supplementary Table 1 - search strategies

Ovid MEDLINE database

	**Oesophageal cancer**
1	(esophag* adj3 adenocarcinoma).mp
2	(oesophag* adj3 adenocarcinoma).mp
3	(esophag* adj3 (neoplasm or cancer or malignan* or carcinoma or tumo?r)).mp
4	(oesophag* adj3 (neoplasm or cancer or malignan* or carcinoma or tumo?r)).mp
5	exp Esophageal Neoplasms/
6	1 OR 2 OR 3 OR 4 OR 5
	**Oesophagectomy concept**
7	esophagectomy.tw
8	oesophagectomy.tw
9	esophageal adj resection.tw
10	oesophageal adj resection.tw
11	surg*.tw
12	7 OR 8 OR 9 OR 10 OR 11
	**Open surgery concept**
13	open.tw
14	standard.tw
15	conventional.tw
16	traditional adj open.tw
17	thoracot*.tw
18	laparot*.tw
19	transthoracic.tw
20	transhiatal.tw
21	13 OR 14 OR 15 OR 16 OR 17 OR 18 OR 19 OR 20
	**Minimally invasive surgery concept**
22	minimally adj invasive.tw
23	thoracoscop*.tw
24	laparoscop*.tw
25	mediastinoscop*.tw
26	robot*.tw
27	single-port.tw
28	multi-port.tw
29	Video-assisted.tw
30	exp Surgical Procedures, Minimally Invasive/
31	MIE.tw
32	MIO.tw
33	22 OR 23 OR 24 OR 25 OR 26 OR 27 OR 28 OR 29 OR 30 OR 31 OR 32
	**RCT concept**

34 exp randomized controlled trial/
35 randomi#ed controlled trial.tw
36 random*.ti,ab,kw
37 trial.ti
38 Random Allocation/
39 (random allocation).tw
40 34 OR 35 OR 36 OR 37 OR 38 OR 39

Combination of concepts

41 6 AND 12 AND 21 AND 33 AND 40

Ovid Embase database

Oesophageal cancer

1 (esophag* adj3 adenocarcinoma).mp
2 (oesophag* adj3 adenocarcinoma).mp
3 (esophag* adj3 (neoplasm or cancer or malignan* or carcinoma or tumo?r)).mp
4 (oesophag* adj3 (neoplasm or cancer or malignan* or carcinoma or tumo?r)).mp
5 exp Esophageal Neoplasms/
6 1 OR 2 OR 3 OR 4 OR 5

Oesophagectomy concept

7 esophagectomy.tw
8 oesophagectomy.tw
9 esophageal adj resection.tw
10 oesophageal adj resection.tw
11 surg*.tw
12 7 OR 8 OR 9 OR 10 OR 11

Open access concept

13 open.tw
14 standard.tw
15 conventional.tw
16 traditional adj open.tw
17 thoracot*.tw
18 laparot*.tw
19 transthoracic.tw
20 transhiatal.tw
21 13 OR 14 OR 15 OR 16 OR 17 OR 18 OR 19 OR 20

Minimally invasive access concept

22 minimally adj invasive.tw
23 thoracoscop*.tw
24 laparoscop*.tw
25 mediastinoscop*.tw

26 robot*.tw
27 single-port.tw
28 multi-port.tw
29 Video-assisted.tw
30 exp Surgical Procedures, Minimally Invasive/
31 MIE.tw
32 MIO.tw
33 22 OR 23 OR 24 OR 25 OR 26 OR 27 OR 28 OR 29 OR 30 OR 31 OR 32

RCT concept

34 exp randomized controlled trial/
35 randomi#ed controlled trial.tw
36 random*.ti,ab,kw
37 trial.ti
38 Random Allocation/
39 (random allocation).tw
40 34 OR 35 OR 36 OR 37 OR 38 OR 39

Combination of concepts

41 6 AND 12 AND 21 AND 33 AND 40

CENTRAL database

Oesophageal cancer

1 (esophag* near/3 adenocarcinoma):ti,ab,kw
2 (oesophag* near/3 adenocarcinoma):ti,ab,kw
3 (esophag* near/3 (neoplasm or cancer or malignan* or carcinoma or tumo?r))
4 (oesophag* near/3 (neoplasm or cancer or malignan* or carcinoma or tumo?r))
5 [mh "Esophageal Neoplasms"]
6 {OR #1-#5}

Oesophagectomy concept

7 esophagectomy
8 oesophagectomy
9 "esophageal resection"
10 "oesophageal resection"
11 surg*
12 {OR #7-#11}

Open access concept

13 open
14 standard
15 conventional
16 "traditional open"
17 thoracot*

18 laparot*
19 transthoracic
20 transhiatal
21 {OR #13-#20}

Minimally invasive access concept

22 "minimally invasive"
23 thoracoscop*
24 laparoscop*
25 mediastinoscop*
26 robot*
27 single-port
28 multi-port
29 Video-assisted
30 [mh "Surgical Procedures, Minimally Invasive"]
31 MIE
32 MIO
33 {OR #22-#32}

Combination of concepts

34 #6 and #12 and #21 and #33

Limits

35 Limit 34 to trials

Supplementary Table 2. Summary of details extracted from each RCT to inform judgments across the PRECIS-2 domains[1]

Domain	Description
**Eligibility**	Verbatim criteria used to include or exclude patients from the trials, grouped by theme where appropriate (e.g., tumour location). Themes developed iteratively between the team.
**Recruitment**	Information regarding the type of centre (clinic, hospital department) where patients were recruited. Any strategies used to recruit patients to the trial, such as examining medical records or advertising.
**Setting**	Information regarding the setting of the trial, such as number, type and location of centres.
**Organisation**	Numbers of open esophagectomy and MIE procedures performed by individual surgeons, participating centres, and surgical teams. Relevant text regarding level of experience required to take part in the trial, such as the number of procedures performed.
**Flexibility: delivery**	Any strategies that aimed to standardise surgical interventions, such as delivery of the interventions by the same surgeon/surgical team or prescriptive protocols.
**Flexibility: adherence**	Any strategies to ensure fidelity to the intervention, such as videorecording.
**Follow-up**	Details about the timing and frequency of follow-up visits, and additional data collected.
**Primary outcome**	Type of outcome, e.g., clinical or patient-reported, and its pertinence to patients.
**Primary analysis**	Ascertaining how data were analysed in the event of crossovers or protocol deviations, information regarding intention-to-treat (ITT) or per protocol analysis (or both).

Supplementary Table 3. Method for the assessment of internal validity using the risk of bias (ROB) 2 tool[2]

Domain	Description
**Randomisation process**	Information about the generation of the random sequence and allocation concealment were extracted, in addition to determination of any baseline difference between intervention groups.
**Deviation from intended interventions**	Reporting of blinding of participants and surgeons was extracted. Information referring to crossovers and protocol deviations was also extracted. Crossovers included both planned and unplanned crossovers between trial groups e.g., MIE to open or vice versa. Protocol deviations were defined as instances when patients either did not receive the intervention due to unforeseen circumstances (e.g., disease progression) or underwent an additional step or component not outlined in the protocol (e.g., additional resection due to suspicious findings).
**Missing outcome data**	Trial results were reviewed to assess the comprehensiveness of the data.
**Measurement of outcome data**	Primary outcomes were extracted, including measures used and timing of last measurement. If a primary outcome was not explicitly defined, the outcome used to calculate sample size was extracted, or if this was not available the first outcome reported in the abstract. If a composite outcome was reported (e.g., complications), all variables contributing to the composite outcome were extracted. Reporting of blinding of outcome assessors was extracted. If outcome assessors were not blinded, any likely influence on the assessment of the outcome was considered (e.g., patient-reported outcome or a blood test)
**Selection of reported results**	Protocols (where available) and pre-specified statistical plans were examined.

Supplementary Table 4. Mean domain scores for PRECIS-2 assessments for each trial.

	PRECIS-2 domains									
**First author**	**Eligibility**	**Recruitment**	**Setting**	**Organisation**	**Flexibility: delivery**	**Flexibility: adherence**	**Follow-up**	**Primary outcome**	**Primary analyses**	**Mean score (trial)**
Biere[3]	4	5	5	4	3	3	3	4	5	4.0
Guo[4]	5	5	2	NR	4	NR	4	2	NR	3.7
Hong[5]	3	NR	2	4	4	NR	2	4	NR	3.2
Paireder[6]	4	5	2	3	3	NR	5	4	1	3.4
Ma[7]	2	5	2	NR	NR	NR	NR	4	NR	3.3
van der Sluis[8]	4	5	2	4	4	NR	4	4	5	4.0
Mariette[9]	4	NR	5	4	2	3	3	4	5	3.8
Zhang[10]	4	4	2	NR	NR	NR	5	2	NR	3.4
Yu[11]	5	5	2	NR	2	NR	NR	2	NR	3.2
**Mean score (domain)**	3.9	4.9	2.7	3.8	3.1	3.0	3.7	3.3	4.0	

NR = not reported

Pragmatic

Equally pragmatic and explanatory

Explanatory

Supplementary Table 5. Verbatim text used to support risk of bias (ROB) judgment using the RoB 2 tool[2] for included studies

Author	ROB domain	RoB judgment	Text to support judgment	
**Biere[3]**	*Randomisation process*	Low risk	From paper	"Randomization is performed per center by an internet randomization module maintained by coordinators at the VUmc. As some heterogeneity is expected, e.g. difference in type of neoadjuvant therapy protocol, randomization will be stratified for each center"
			To note	[There is no explicit mention of allocation concealment, however the use of an internet-based randomization programme would suggest allocation was done by the software] (KAC)
	Deviations from intended interventions	Low risk	From paper	"Patients, and investigators undertaking interventions, assessing outcomes, and analysing data were not masked to group assignment"<br>"2 refused open surgery and underwent MIO"<br>"Four crossovers occurred: two patients assigned to the open oesophagectomy group underwent minimally invasive oesophagectomy, and two assigned to minimally invasive oesophagectomy developed a WHO-ECOG score of 3 during neoadjuvant treatment and thus had transhiatal oesophagectomy. Eight patients did not undergo a resection; we included these patients in the analysis of the allocated group"<br>"Analysis was by intention to treat"
			To note	NA
	Missing outcome data	Low risk	From paper	"56 patients were analysed in the open oesophagectomy group and 59 in the minimally invasive group"
			To note	[Data were available for all patients] (KAC)
	Measurement of the outcome	Low risk	From paper	"The primary outcome was postoperative pulmonary infection, defined as clinical manifestation of pneumonia or bronchopneumonia confirmed by thoracic radiographs or CT scan (assessed by independent radiologists) and a positive sputum culture"<br>"Patients, and investigators undertaking interventions, assessing outcomes, and analysing data, were not masked to group assignment"
			To note	[It is unlikely that assessment of the outcome could have been influenced by knowledge of intervention received] (KAC)
	Selection of the reported result	Low risk	From paper	"…data analysis will be performed in accordance with the intention-to-treat principle, additional per-protocol analysis will also be performed. Groups are, where appropriate, compared using an Independent Samples T-test, otherwise a Wilcoxon test, or Chi-square test"

				"…pulmonary infection, defined as clinical manifestation of pneumonia or bronchopneumonia confirmed by thoracic radiographs or CT scan assessed by independent radiologists) and a positive sputum culture"
			To note	[The protocol was available. Data reported were in line with that specified in protocol and the outcomes stated in the protocol and paper were all reported] (KAC)
	Overall risk of bias	Low risk		
**Guo[4]**	*Randomisation process*	Some concerns	From paper	"…prospective randomized in two groups"<br>"The 221 patients were divided into two groups" (Table 1)
			To note	[There was no information about the randomisation sequence and no information regarding allocation concealment. The baseline data are sparse but there doesn't look to be any substantial differences between the two groups] (KAC)
	Deviations from intended interventions	High risk	From paper	NA
			To note	[There was no information about blinding of patients, carers or people delivering the intervention, though due to the nature of the procedure, patients and surgeons were likely to have been aware of intervention allocation. There was also no information in the text, table or figures to indicate the numbers for each intervention group, so it is unclear whether there were any deviations from intended intervention. There was also no information about intention-to-treat analysis.] (KAC)
	Missing outcome data	Some concerns	From paper	NA
			To note	[There was no information in the text, tables or figures to indicate that any outcome data were missing. The outcome is 'length of operation' and there is no information in the text or tables to indicate that anyone did not receive surgery] (KAC)
	Measurement of the outcome	High risk	From paper	NA
			To note	[There was no information regarding the method used to assess the outcome (length of operation) or whether the outcome assessor was blinded. Since the outcome is 'length of operation', a timing device must have been used and most likely assessed by someone observing the surgical procedure. It is unlikely that the surgeons would have been blinded therefore they could conceivably have slowed or sped up the operation in order to affect the length of operation] (KAC)
	Selection of the reported result	Some concerns	From paper	NA
			To note	[There was no protocol or clinical trial registry entry to indicate the intended primary outcome or statistical plan. Length of operation would not have been selected from multiple outcome measurements or multiple analyses] (KAC)

	Overall risk of bias	High risk		
**Hong[5]**	*Randomisation process*	Some concerns	From paper	"…randomly assigned by a computer-generated randomization sequence, in a 1:1 ratio" "The demographics and clinical characteristics of the two groups were similar at baseline. Age, sex, smoking history, body mass index, weight loss, ASA classification, differentiation status, and tumor stage were similarly distributed between the groups".
			To note	[There was no information regarding allocation concealment] (KAC)
	Deviations from intended interventions	Some concern	From paper	NA
			To note	[There was no information about blinding of patients, carers or people delivering the intervention, though due to the nature of the procedure, patients and surgeons were likely to have been aware of intervention allocation. There was no information in the text, table or figures to indicate that there were any deviations from intended intervention. There was also no information about intention-to-treat analysis. However, this study was retrospective, and it seems that following randomisation and surgery, patients were excluded according to specific criteria. They may not have included any patients that were converted to the other intervention, though this is not explicitly reported] (KAC)
	Missing outcome data	Low risk	From paper	NA
			To note	[There was no information in the text about missing data. Table 2 suggested that all data were complete] (KAC)
	Measurement of the outcome	Some concerns	From paper	NA
			To note	[There was no information regarding the method used to assess the outcome (pulmonary infection) or whether the outcome assessor was blinded. It is unlikely that the outcome would have been influenced by knowledge of the intervention] (KAC)
	Selection of the reported result	Some concerns	From paper	NA
			To note	[There was no protocol or clinical trial registry entry to indicate the intended primary outcome or statistical plan. Pulmonary complication rate could have been selected from multiple outcome measurements or multiple analyses, but this is unclear] (KAC)
	Overall risk of bias	Some concerns		
**Paireder[6]**	*Randomisation process*	Low risk	From paper	"A computer-based online randomizing tool, provided by the Medical University of Vienna, was used to perform randomization" "Randomization was performed by the study center"

			To note	[There is no explicit mention of allocation concealment, however the use of an internet-based randomization programme would suggest allocation was done by the software] (KAC)
	Deviations from intended interventions	Some concerns	From paper	"Three (11.5%) patients dropped out due to progression of disease and merely underwent explorative surgery. Finally, 26 (20 male, 76.9%) patients underwent surgery after randomization."
			To note	[There was no information about blinding of patients, carers or people delivering the intervention. There was also no information about intention-to-treat analysis] (KAC)
	Missing outcome data	Low risk	From paper	NA
			To note	[There was no information in the text about missing data. Table 3 suggested that all data were complete] (KAC)
	Measurement of the outcome	Low risk	From paper	"Morbidity was grouped in Clavien/Dindo (C/D) classification. C/D grades I & II were considered as minor complications, C/D grades I & II were considered as minor complications, III a,b and IV a,b were referred to as major complications"
			To note	[There was no information about blinding of the outcome assessors. It is unlikely that the outcome would have been influenced by knowledge of the intervention as the outcome is not subjective] (KAC)
	Selection of the reported result	Some concerns	From paper	NA
			To note	[There was no protocol to indicate the intended primary outcome or statistical plan. The entry on the clinical trial database was added after the study was completed and stated that morbidity would be assessed by measuring the frequency of anastomotic leakage, gastric conduit necrosis and/or pneumonia. In the main paper, Clavien/Dindo classification was the primary outcome] (KAC)
	Overall risk of bias	Some concerns		
**Ma[7]**	*Randomisation process*	Some concerns	From paper	"…were randomly selected to treat by one of two teams of surgeons with a preference for either OE or MIE"<br>"Baseline demographics and clinicopathologic characteristics, including age, gender, SI, pulmonary function, BMI, ASA score, tumor type and location, and final pathological TNM stage of the two groups were comparable [Table 1]. None of these characteristics was with obvious statistical significance between the two groups"
			To note	[There was no information regarding allocation concealment]<br>[97 patients underwent open and 47 MIO. There was no information to suggest a 2:1 randomisation was planned] (KAC)

	Deviations from intended interventions	Some concerns	From paper	"Two patients who were first assigned to the minimally invasive group conversed to thoracotomy because local infiltration and intraoperative hemorrhage could not be controlled under the thoracoscopic procedure"
			To note	[There was no information about blinding of patients, carers or people delivering the intervention. There was also no information about intention-to-treat analysis] (KAC)
	Missing outcome data	Low risk	From paper	NA
			To note	[There was no information in the text about missing data. Table 3 suggested that all data were complete] (KAC)
	Measurement of the outcome	Low risk	From paper	"The Comprehensive Complication Index (CCI) was first developed in 2013 by Slankamenac *et al*.,[20] aiming to integrates all the negative events with their respective severity. The CCI is calculated as the sum of all complications with different severities classified by CDC which may ignore all additional complications that are equal or less severe. The CCI values range from 0 to 100; a value of 0 reflects the absence of complications, while a CCI of 100 indicates that the patient has died due to the occurrence of the complications. Several kinds of literature had confirmed that the CCI was a better parameter to identify risks in surgical patient groups"
			To note	[There was no information about blinding of outcome assessors. It is unlikely that the outcome would have been influenced by knowledge of the intervention as the outcome is not subjective] (KAC)
	Selection of the reported result	Some concerns	From paper	NA
			To note	[There was no protocol or clinical trial registry entry to indicate the intended primary outcome or statistical plan. However, the primary outcome assessed does not appear to have been selected on the basis of results from multiple outcome measurements or multiple analyses] (KAC)
	Overall risk of bias	Some concerns		
**van der Sluis[8]**	*Randomisation process*	Low risk	From paper	"Allocation of concealment was performed using computer generated random numbers in sealed opaque envelopes corresponding to either RAMIE or OTE"<br>"Demographic and clinical characteristics were similar at baseline"
			To note	[There is no information about the envelopes being sequentially numbered; we do not consider this enough to rate this domain as 'some concern'] (KAC)
	Deviations from intended interventions	Low risk	From paper	"Operator blinding for the procedure was not possible"<br>"There is no blinding for the patient, surgeon and coordinating researcher because this is difficult in daily practice. However, the independent data monitoring safety committee is blinded to the allocated intervention"<br>"One crossover occurred: 1 patient assigned to the OTE group underwent RAMIE due to a WHO-ECOG score of 3 after neoadjuvant treatment. In the RAMIE group, 2 patients were found to have

				irresectable disease intraoperatively. In 1 patient tumor ingrowth to the aorta was found and in 1 patient liver metastases were discovered intraoperatively. All these patients were included in the ITT analysis”
			To note	NA
	Missing outcome data	Low risk	From paper	NA
			To note	[There was no information in the text about missing data. Table 2 suggested that all data were complete] (KAC)
	Measurement of the outcome	Low risk	From paper	“The primary endpoint of this study was the percentage of overall surgery-related postoperative complications modified Clavien-Dindo classification (MCDC) surgical complications grade ≥ 2” “Outcomes were discussed in a weekly multidisciplinary meeting, where the participants were unaware of treatment allocation”
			To note	[It is not clear as to who the outcome assessors were, so they may or may not have been blinded. It is unlikely that the outcome would have been influenced by knowledge of the intervention as the outcome is not subjective] (KAC)
	Selection of the reported result	Low risk	From paper	“Data analysis will be performed in accordance with the intention-to-treat principle; To evaluate significance of differences between the two groups, chi-squared and Fisher's exact tests will be used as appropriate for categorical variables, and the nonparametric Mann–Whitney U test for continuous variables” “Results are presented as risk ratios (RRs) with corresponding 95% confidence intervals (CIs). To evaluate significance of differences between groups, the χ2 test was used as appropriate for categorical variables and the Student T test and nonparametric Mann-Whitney U test for continuous variables”
			To note	[The protocol was available. Data reported were in line with that specified in protocol and the outcomes stated in the protocol and paper were all reported; however, the result was presented as a risk ratio (RR) which was not mentioned in the protocol. However we do not feel this would affected the analyses] (KAC)
	Overall risk of bias	Low risk		
**Mariette[9]**	*Randomisation process*	Some concerns	From paper	“Randomization was performed centrally, with the use of the stratified-field block-randomization method (blocks of four) for each participating center. A randomization list was generated for each center, and numbered envelopes were prepared” “The blinded assignment to a trial group was done during surgery, according to serial inclusion”
			To note	[Block of randomisation of four is quite small and could enable surgeons to know what surgery is due next and could influence who they enrol in the trial next. However, if the surgeon and theatre staff were unaware of the size of the block being used then there would not be any risk of bias.

				Details about the envelopes were lacking – they were not reported to be *sequential* numbered, opaque, or sealed] (KAC)
	Deviations from intended interventions	Low risk	From paper	"Patients, physicians, and investigators were aware of the assigned treatment group during or immediately after surgery"<br>"A total of 3 patients (3%) who had been assigned to the hybrid-procedure group underwent intraoperative conversion to the open procedure: 1 underwent laparotomy without resection because of advanced disease, 1 underwent intraoperative conversion to the open procedure because of subcutaneous emphysema, and 1 underwent intraoperative conversion to the open procedure, as decided by the surgeon on the basis of intraoperative physiological stress of the patient. According to the intention-to-treat principle, these patients were included in the hybrid-procedure group"
			To note	NA
	Missing outcome data	Low risk	From paper	NA
			To note	[There was no information in the text about missing data. Table 2 suggested that all data were complete] (KAC)
	Measurement of the outcome	Low risk	From paper	"A major intraoperative and postoperative complication was defined as a surgical or medical complication with a Clavien–Dindo grade of II or higher (this five-grade system includes subgrades in grades III and IV, and higher grades indicate more life-threatening complications). The most severe complication in a patient was considered for the classification of the primary end point"<br>"Patients, physicians, and investigators were aware of the assigned treatment group during or immediately after surgery"<br>"According to the intention-to-treat principle, these patients were included in the hybrid-procedure group"
			To note	[The outcome assessors were not blinded; however, it is unlikely that the outcome would have been influenced by knowledge of the intervention as the outcome is not subjective] (KAC)
	Selection of the reported result	Low risk	From paper	"Major morbidity is defined as Clavien grade 2 complications (potentially life-threatening complications without residual disability), grade 3 complications (potentially life-threatening complications with residual disability) and grade 4 complications (death) (Appendix 12), and will be assessed by counting the number of patients presenting with one or more complications. The total numbers of complications and each grade will be specified. When assessing the primary criterion, we shall take account of major complications occurring before surgery and during the 30 days following surgery"
			To note	[The protocol was available. Data reported were in line with that specified in protocol and the outcomes stated in the protocol and paper were all reported] (KAC)

	Overall risk of bias	Some concerns		
**Zhang[10]**	*Randomisation process*	Some concerns	From paper	“One hundred elderly patients with esophageal cancer hospitalized in Renmin Hospital, Hubei University of Medicine, from June 2014 to June 2016 were divided into two equal groups (50 patients per group) with a random number table, namely, a control group and an observation group”<br>“There were no significant differences between the two groups in terms of these general characteristics (p>0.05).”
			To note	[There was no information regarding allocation concealment. There was no table showing baseline data but authors reported there were no statistical differences between the two groups] (KAC)
	Deviations from intended interventions	High risk	From paper	NA
			To note	[There was no information about blinding of patients, carers or people delivering the intervention. There was no information in the text, table or figures to indicate that there were any deviations from intended intervention. There was also no information about intention-to-treat analysis] (KAC)
	Missing outcome data	Low risk	From paper	NA
			To note	[There was no information in the text about missing data. Table I suggested that all data were complete] (KAC)
	Measurement of the outcome	High risk	From paper	NA
			To note	[There was no information regarding the method used to assess the outcome (length of operation) or whether the outcome assessor was blinded. Since the outcome is ‘length of operation’, a timing device must have been used and most likely assessed by someone observing the surgical procedure. It is unlikely that the surgeons would have been blinded therefore they could conceivably have slowed or sped up the operation in order to affect the length of operation] (KAC)
	Selection of the reported result	Some concerns	From paper	NA
			To note	[There was no protocol or clinical trial registry entry to indicate the intended primary outcome or statistical plan. Length of operation would not have been selected from multiple outcome measurements or multiple analyses] (KAC)
	Overall risk of bias	High risk		
**Yu[11]**	*Randomisation process*	Some concerns	From paper	“randomly selected for prospective analysis, and were divided equally and randomly into a control group and an experimental group of 45 patients each according to the time of admission”

			To note	[There was no information about the randomisation sequence and no information regarding allocation concealment] (KAC)
	Deviations from intended interventions	Some concerns	From paper	NA
			To note	[There was no information about blinding of patients, carers or people delivering the intervention, though due to the nature of the procedure, patients and surgeons were likely to have been aware of intervention allocation. There was no information in the text or tables to indicate that there were any deviations from intended intervention.] (KAC)
	Missing outcome data	Low risk	From paper	NA
			To note	[There was no information in the text about missing data. Tables 2-4 suggested that all data were complete] (KAC)
	Measurement of the outcome	High risk	From paper	NA
			To note	[There was no information regarding the method used to assess the outcome (length of operation) or whether the outcome assessor was blinded. Since the outcome is ‘length of operation’, a timing device must have been used and most likely assessed by someone observing the surgical procedure. It is unlikely that the surgeons would have been blinded therefore they could conceivably have slowed or sped up the operation in order to affect the length of operation] (KAC)
	Selection of the reported result	Some concerns	From paper	NA
			To note	[There was no protocol or clinical trial registry entry to indicate the intended primary outcome or statistical plan. Length of operation would not have been selected from multiple outcome measurements or multiple analyses] (KAC)
	Overall risk of bias	High risk		

References

1. Loudon K, Treweek S, Sullivan F, et al. The PRECIS-2 tool: designing trials that are fit for purpose. *BMJ* 2015;350:h2147. doi: 10.1136/bmj.h2147
2. Sterne JAC, Savović J, Page MJ, et al. RoB 2: a revised tool for assessing risk of bias in randomised trials. *BMJ* 2019;366:l4898. doi: 10.1136/bmj.l4898
3. Biere S, Berge HM, Maas K, et al. Minimally invasive versus open oesophagectomy for patients with oesophageal cancer: a multicentre, open-label, randomised controlled trial. *Lancet (London, England)* 2012;379(9829):1887-92. doi: https://doi.org/10.1016/S0140-6736(12)60516-9
4. Guo M, Xie B, Sun X, et al. A comparative study of the therapeutic effect in two protocols: video-assisted thoracic surgery combined with laparoscopy versus right open transthoracic esophagectomy for esophageal cancer management. *Chinese-German Journal of Clinical Oncology* 2013;12(2):P68-p71. doi: https://doi.org/10.1007/s10330-012-0966-0
5. Hong L, Zhang Y, Zhang H, et al. The short-term outcome of three-field minimally invasive esophagectomy for Siewert type I esophagogastric junctional adenocarcinoma. *Ann Thorac Surg* 2013;96(5):1826-31. doi: https://doi.org/10.1016/j.athoracsur.2013.06.058
6. Paireder M, Asari R, Kristo I, et al. Morbidity in open versus minimally invasive hybrid esophagectomy (MIOMIE): long-term results of a randomized controlled clinical study. *European Surgery - Acta Chirurgica Austriaca* 2018;50(6):249-55. doi: https://doi.org/10.1007/s10353-018-0552-y
7. Ma G, Cao H, Wei R, et al. Comparison of the short-term clinical outcome between open and minimally invasive esophagectomy by comprehensive complication index. *J Cancer Res Ther* 2018;14(4):789-94. doi: https://doi.org/10.4103/jcrt.JCRT_48_18
8. van der Sluis PC, van der Horst S, May AM, et al. Robot-assisted Minimally Invasive Thoracolaparoscopic Esophagectomy Versus Open Transthoracic Esophagectomy for Resectable Esophageal Cancer: A Randomized Controlled Trial. *Ann Surg* 2019;269(4):621-30. doi: https://doi.org/10.1097/sla.0000000000003031
9. Mariette C, Markar SR, Dabakuyo-Yonli TS, et al. Hybrid Minimally Invasive Esophagectomy for Esophageal Cancer. *N Engl J Med* 2019;380(2):152-62. doi: 10.1056/NEJMoa1805101
10. Zhang T, Jiang H, Ming CY, et al. Influence of different kinds of surgical resection on operation-related clinical indexes, inflammatory cytokines and complications in elderly patients with esophageal cancer. *Pakistan Journal of Medical Sciences* 2020;36(3):532-37. doi: 10.12669/pjms.36.3.1465
11. Yu Y, Han Y. Clinical Effect and Postoperative Pain of Laparo-Thoracoscopic Esophagectomy in Patients with Esophageal Cancer. *Evid Based Complement Alternat Med* 2022;2022:4507696. doi: https://dx.doi.org/10.1155/2022/4507696
