# Supplementary material for: How pragmatic are randomised controlled trials evaluating minimally invasive surgery for oesophageal cancer? A methodological review of trial design using the Pragmatic-Explanatory Continuum Indicator Summary-2 (PRECIS-2) tool
Source: BMJ Open. 2024 Dec 20;14(12):e078417. doi: 10.1136/bmjopen-2023-078417 (PMC11664389; doi:10.1136/bmjopen-2023-078417)
Supplement: Supplementary file 2 [file bmjopen-2023-078417.reviewer_comments.pdf]

PEER REVIEW HISTORY

BMJ Open publishes all reviews undertaken for accepted manuscripts. Reviewers are asked to complete a checklist review form and are provided with free text boxes to elaborate on their assessment. These free text comments are reproduced below.

ARTICLE DETAILS

Title (Provisional)

How pragmatic are randomised controlled trials evaluating minimally invasive surgery for oesophageal cancer? A methodological review of trial design using the PRECIS-2 tool

Authors

Chalmers, Katy; Cousins, Sian; Blencowe, Natalie S; Blazeby, Jane

---

VERSION 1 - REVIEW

**Reviewer**	**1**
**Name**	**Reynolds, John**
**Affiliation**	**Trinity College Dublin**
**Date**	**09-Oct-2023**
**COI**	**none**

This detailed analysis is valuable to the oesophageal community, in particular surgeons, in highlighting significant deficits in trial design. It is ironic that the one RCT of highest quality with low risk of bias could not be externally validated (J Clin Oncol 2020; 38: 2130-9), and this apparent discordance should be teased out further in the Discussion.

**Reviewer**	**2**
**Name**	**Bach, Simon**
**Affiliation**	**Queen Elizabeth Hospital Birmingham**
**Date**	**24-Oct-2023**
**COI**	**N/a**

Introduction: The premise is clear; how do we approach achieving balance in design and conduct of RCTs that evaluate complex interventions such as surgery in order to achieve results that are not only true in the context of the study, but also may be widely applicable in clinical practice. Here the authors consider the validity and applicability of RCTs evaluating open versus minimally invasive oesophagectomy.

Methods: Searches for relevant research papers were conducted anually (3 occasions) to inform progress of the ROMIO RCT. Relevant studies were evaluated for validity and pragmatism using Risk of Bias 2 (developed by the same group) and PRECIS-2 tools respectively. Some licence was taken in assimilating domain information from PRECIS 2 to form overall study scores that defined RCTs as categorical variable i.e., explanatory versus pragmatic. In addition, presence of a detailed specification for the surgical intervention in the study protocol was used as a proxy for measurement of adherence to the trial intervention (as a component of PRECIS 2).

Results: 9 RCTs comparing open versus MIO were included. 5/9 single centre and only 1 international. PRECIS 2 assessment was incomplete for 8/9 studies (due to lack of published information). Commonly missing information related to adherence to the intervention and the extent of data ascertainment for analysis. Risk of bias was evaluated for every study, 3/9 scored high risk in multiple domains.

Comments:

1. The paper deals with categorizing RCTs of MIO as explanatory versus pragmatic. For the most part the studies identified were single centre, low number of participants. Primary outcomes ranged from quality of life to overall survival. On the face of it these studies are likely to have had substantial methodological challenges relating to learning curve, power, and the effect sizes usd to calculate sample sizes. I believe that these parameters are not directly assessed by the RoB2 tool. It would be interesting to know what proportion of studies (i) set realistic effect sizes and (ii) were adequately powered to detect a difference between the two treatments?

2. Would the authors like to comment on whether a 'trial vehicle' needs to have secure statistical foundations for the categorisation of explanatory versus pragmatic to be deemed of real value? For example, is it not possible that the 'explanatory' categorisation might otherwise encompass/ become a surrogate for a disproportionate number of poorly designed or underpowered studies?

2. I note that delivery of the trial intervention was not assessable in the context of PRECIS 2 for the majority of studies, however, this also forms a component of RoB2 scoring. I note that there was poor consensus (37%) for initial RoB2 scoring of deviations from the intended interventions. It is not clear why one assessment was not possible whereas the other was; albeit with poor consensus. Why was consensus so poor and how was this resolved?

3. In the context of studies evaluating complex interventions that are likely to incorporate a learning curve for at least one arm, to what extent can the distinction between exploratory versus pragmatic study be overcome by simply presenting ITT (treatment allocated) and mITT (treatment delivered as intended) analysis alongside each other? Is it possible to design a single study that is able to present results from both perspectives?

Reviewer 3

Name Zhang, Peng

Affiliation Capital Medical University Affiliated Beijing Friendship Hospital

Date 18-Jan-2024

COI None

The submitted manuscript assessed the applicability and validity of RCTs involving the comparison between minimally invasive and open esophageal surgery by using PRECIS-2 and Risk-of-Bias 2 tools. The results showed that only one out of nine trials was able to be considered as “pragmatic”. Therefore, the authors recommended that the intended purpose of a RCT (explanatory or pragmatic) should be clear when designing the trial.

Overall, the objectives of this review were well defined, the assessment tools and methodologies were properly adopted, the 9 involved RCT reports were thoroughly reviewed and analyzed, the results were meaningful, and therefore, the conclusion from this review was unbiased, validated and clinically applicable. The reviewer still has a few minor questions for the authors:

1) Regarding the assessment of pragmatism with PRECIS-2 tool, each domain was scored from 1 to 5, were there any “objective” standardized criteria to define a score of 2, 3 or 4 for there specific trials? Can the authors disclose which domains were not agreed initially by the two researchers, but why did one researcher accept the other’s assessment later? Was a thoracic surgeon specialist invited to the discussion when the two independent researchers did not reach agreement on scores initially?

2) The reviewer agree that the trial needs to be pragmatic if aiming to change practice, or need to be explanatory if establishing mechanisms. However, can the authors discuss more on which conclusion should be more reliable if paradoxical conclusions were drawn from a pragmatic trial and an explanatory trial for the same clinical hypothesis, and why?

Reviewer 4

Name Chambers, David

Affiliation National Cancer Institute

Date 20-Feb-2024

COI I have no competing interests.

This is a well-written, exceptionally clear paper that furthers along the conversation about how best to apply available tools to assess the pragmatic vs. explanatory nature of trials. It breaks new ground by applying these tools to surgical interventions, through a review of a specific type of trial, and offers important commentary on the challenges of using the tool,

the implication of findings, and potential next steps for the field to consider. The background, methodology, results and discussion are all quite succinct and accessible to the average reader, and the points raised in the review underscore the important issues of applying tools created to assist in design of trials to the summation of trials in a given field.

The article acknowledges limitations inherent in the study, particularly related to the limited information provided in publications to determine appropriate ratings, the particularities of surgical interventions and how they should be interpreted according to specific domains, and the lack of existing guidance on defining a line between exploratory and pragmatic. All of this is handled by the authors extremely well.

There is one particular area that I would suggest would be useful to expand in the discussion. Critical assessment in each domain of the impact of a pragmatic vs. explanatory score might lead to an ability to weight the different domains in terms of deriving a summary score versus the mean. I would appreciate the authors' insights on whether certain domains are naturally less relevant for specific types of studies or interventions, and they would suggest rating a subset of domains or more heavily weighing some versus others. Implications of a pragmatic score in a domain that may run counter to the type of intervention would be useful for the field to learn and could be done given the team's analysis and expertise. While the authors suggest that better guidance on scoring would be useful, I would appreciate understanding their guidance, given the experience of completing this study. The use of the Risk-of-Bias 2 tool is additive as well, although the study might benefit from more integration (including in the title) of how the PRECIS-2 and ROB-2 tools complement one another. Right now, it seems like the risk of bias aspects of the study are disconnected from the overall discussion.

With consideration of these points, I see the resulting article as one of benefit to the trials community and look forward to seeing a revised discussion should it be needed.

**Reviewer**	**5**
**Name**	**Thorpe, Kevin**
**Affiliation**	
**Date**	**21-Feb-2024**
**COI**	**None.**

The paper is generally well written. However, I believe if proceeds from a false premise; that pragmatic trials are preferred over explanatory. Therefore, my comments will focus more on context.

First, there appears to be a desire to have cut-offs to declare a trial as pragmatic. This, in fact, is completely contrary to why PRECIS and PRECIS-2 were designed. Due to the multidimensional factors it seemed overly simplistic to reduce trials to a binary classification.

The domains actually matter. The push for a classification implies a belief that trials of more pragmatic character are preferred over ones tending to explanatory. I disagree with this.

Second, there is an important characteristic of PRECIS-2 that I believe is frequently overlooked. When PRECIS-2 was developed, the lead authors approached it from the standpoint of developing the tool to help design pragmatic trials. This can be seen from the absence of domains related to the comparator group. In PRECIS-2 the comparator group is assumed to be usual care. The paper does indicate that if your comparator is not usual care you should also perform the PRECIS-2 assessment on it. This becomes very important in light of the first sentence of the discussion which states, "... comparing open versus minimally invasive oesophagectomy to usual clinical practice." The final words of this sentence imply there is also a usual practice that is not reflective of either of the treatment groups considered in the trials reviewed. If my interpretation is correct and the trials reviewed were claimed to be pragmatic by their authors, the situation may be even more grim than the authors of the present paper report.

One minor point worth raising is that of internal validity, as mentioned by the authors. It is important to keep in mind that internal validity considerations for an explanatory leaning trial should not be applied to the pragmatic leaning trial since the goals are different.

The tendency I have seen in trials is to call them pragmatic in those cases where the investigator wishes deliver the interventions as they would in practice, However, without careful consideration of all design elements, the generalizability hoped for is likely to suffer. I think the authors have done a good job at showing the apparent lack of attention to the other domains.

In summary, I think this paper could be greatly strengthened by considering what kinds of trials are needed in this field and how the trials reviewed address or fail to address those needs. This is in addition to addressing the two main points above.

I hope the authors find my comments helpful and thought-provoking.

VERSION 1 - AUTHOR RESPONSE

Reviewer 1

Dr. John Reynolds, Trinity College Dublin

Comments to the Author:

This detailed analysis is valuable to the oesophageal community, in particular surgeons, in highlighting significant deficits in trial design. It is ironic that the one RCT of highest quality with low risk of bias could not be externally validated (J Clin Oncol 2020; 38: 2130-9), and this apparent discordance should be teased out further in the Discussion.

Response:

Thank you for comments about the discordance between our assessments and the reported lack of applicability of the TIME trial to clinical practice in the Netherlands. In our study, the TIME trial (Biere et al) was found to have good internal validity (low risk of bias) according to the Cochrane ROB 2 tool. Our assessment for external validity using the PRECIS-2 tool, which addresses the applicability (generalisability) of the trial to clinical practice, was comprehensive (ie. all domains were assessed) suggesting that overall, the trial had more pragmatic features than explanatory. The validation study undertaken by Markar et al. suggested that the TIME trial was not applicable (generalisable) to clinical practice for these main reasons: 1) the patient population was different to the TIME trial – national practice assessed in the audit data showed patients had different ASA grades and pre-operative tumour staging; and 2) the surgeons undertaking MIO were less experienced that the surgeons that participated in the TIME trial. In our assessment of these specific features, we classified the TIME trial as being 4 (of 5) because Siewert II and III patients were excluded and 3 (of 5) because of the trial's quality assurance program. We could perhaps have considered that the experience required for surgeons to take part in the TIME trial (10 MIOs) was more than what you would expect of a normal surgeon performing an MIO and therefore more explanatory (maybe a 3 instead of a 4). We felt that these procedures were likely to be undertaken in a specialist centre by experienced surgeons, however, Markar et al.'s validity study has shown that surgeons performing this procedure in normal clinical practice in the Netherlands did not have the skill level to perform MIOs effectively. We have added text to reflect on this discordance in the Discussion (page 12, lines 8-12).

Reviewer 2

Mr. Simon Bach, Queen Elizabeth Hospital Birmingham

Comments to the Author:

Introduction: The premise is clear; how do we approach achieving balance in design and conduct of RCTs that evaluate complex interventions such as surgery in order to achieve results that are not only true in the context of the study, but also may be widely applicable in clinical practice. Here the authors consider the validity and applicability of RCTs evaluating open versus minimally invasive oesophagectomy.

Methods: Searches for relevant research papers were conducted annually (3 occasions) to inform progress of the ROMIO RCT. Relevant studies were evaluated for validity and pragmatism using Risk of Bias 2 (developed by the same group) and PRECIS-2 tools respectively. Some licence was taken in

assimilating domain information from PRECIS 2 to form overall study scores that defined RCTs as categorical variable i.e., explanatory versus pragmatic. In addition, presence of a detailed specification for the surgical intervention in the study protocol was used as a proxy for measurement of adherence to the trial intervention (as a component of PRECIS 2).

Results: 9 RCTs comparing open versus MIO were included. 5/9 single centre and only 1 international. PRECIS 2 assessment was incomplete for 8/9 studies (due to lack of published information). Commonly missing information related to adherence to the intervention and the extent of data ascertainment for analysis. Risk of bias was evaluated for every study, 3/9 scored high risk in multiple domains.

Comments:

1. The paper deals with categorizing RCTs of MIO as explanatory versus pragmatic. For the most part the studies identified were single centre, low number of participants. Primary outcomes ranged from quality of life to overall survival. On the face of it these studies are likely to have had substantial methodological challenges relating to learning curve, power, and the effect sizes usd to calculate sample sizes. I believe that these parameters are not directly assessed by the RoB2 tool. It would be interesting to know what proportion of studies (i) set realistic effect sizes and (ii) were adequately powered to detect a difference between the two treatments?

We agree that these studies are likely to have other methodological challenges than those assessed by the PRECIS-2 and ROB2 tools. We have looked at the eligible studies and found that four (Biere, Mariette, Paireder and van der Sluis) had calculated and reported sample sizes which were determined using effect sizes from published literature (Biere, Mariette and Paireder) and/or the authors' own experience/previous work (Biere, Paireder and van der Sluis). All four of these studies had 80% power to detect a difference between the two treatments (α level 0.05). Looking at the results of these studies, three randomised more patients than the calculated sample size (Biere, Mariette and van der Sluis), whilst one fell short (Paireder) and therefore did not have the power to detect any changes in morbidity rates between the two treatments. We have added information in the results section (pg 8, lines 9-10) regarding the studies who reported study power, those who performed sample size calculations and whether or not this was achieved.

2. Would the authors like to comment on whether a 'trial vehicle' needs to have secure statistical foundations for the categorisation of explanatory versus pragmatic to be deemed of real value? For

example, is it not possible that the 'explanatory' categorisation might otherwise encompass/ become a surrogate for a disproportionate number of poorly designed or underpowered studies?

We thank the reviewer for this comment. We consider that all trials need to be designed appropriately according to the research question. This will determine the degree of pragmatism or otherwise of its design. It is possible to create a well-designed and powered study with an explanatory approach provided a good rationale is given for this. The PRECIS-2 tool (whilst created for reporting) is valuable for pragmatic study design. We have updated the discussion to describe this viewpoint (pgs 12 and 13).

2. I note that delivery of the trial intervention was not assessable in the context of PRECIS 2 for the majority of studies, however, this also forms a component of RoB2 scoring. I note that there was poor consensus (37%) for initial RoB2 scoring of deviations from the intended interventions. It is not clear why one assessment was not possible whereas the other was; albeit with poor consensus. Why was consensus so poor and how was this resolved?

Thank you for highlighting this apparent inconsistency. The PRECIS-2 and ROB2 tools both assess whether interventions were delivered as intended but use different approaches. The ROB2 tool provides an Excel-based macro with specific questions (answer choices - yes, probably yes, no, probably no, no information) about blinding of participants, care givers and intervention providers, whether there were any deviations that arose because of experimental context, if they were likely to have an effect the outcome, if they were balanced between groups and if an appropriate analysis was used i.e. intention-to-treat. An algorithm then generates an overall answer for this domain. With the PRECIS-2 tool, the assessments are more subjective. The 'flexibilty: adherence' domain was originally designed to assess patient adherence to the intervention (e.g., taking medication or following therapy), however, since surgical interventions are not delivered by the patient, in this review, as stated in our manuscript, we used this domain to assess the adherence of the surgeon to the protocol (if there was one). With surgical procedures, documentation to report how the surgery was performed such as operation notes/case report forms, would be considered normal (pragmatic), however, additional approaches such as video or audio recording, photographs, checklists, and direct observations might be considered more explanatory. In this review, only two studies reported any form of measuring adherence; the other seven provided no information. Hopefully, this demonstrates that the two tools look at different aspects of intervention delivery and are therefore not comparable.

The reason for poor consensus with the ROB2 scoring of 'delivery of intended interventions' initially, was because of the subjectivity of the answer options in the macro. For example, for the questions about patient blinding, in the absence of any information about blinding, one researcher would select

‘no information’ whilst the other researcher would select ‘probably no’ due to the invasive nature of the procedure and the lack of any methods to disguise surgical wounds. The different answers would result in a different output by the algorithm. The researchers met after having completed a couple of assessments to go through conflicts and the scoring criteria were refined to ensure concordance.

3. In the context of studies evaluating complex interventions that are likely to incorporate a learning curve for at least one arm, to what extent can the distinction between exploratory versus pragmatic study be overcome by simply presenting ITT (treatment allocated) and mITT (treatment delivered as intended) analysis alongside each other? Is it possible to design a single study that is able to present results from both perspectives?

It is agreed that undertaking pre-planned sensitivity analyses (mITT, or excluding participants who did not receive their allocated treatment or did not undergo surgery) can provide an important perspective about the results and their interpretation. The discussion has been updated to highlight this point (page 13).

One method for considering the impact of surgical learning and skill on trial outcomes is to undertake and report pre-agreed sensitivity analyses alongside the main intention to treat analysis. These could i) exclude participants who did not receive the allocated intervention, ii) exclude participants who did not undergo surgery, iii) exclude participants with missing outcome data and iv) analysing by treatment received. This provides an opportunity to consider the impact of these different analyses of trial findings.

Reviewer: 3
Prof. Peng Zhang, Capital Medical University Affiliated Beijing Friendship Hospital
Comments to the Author:
The submitted manuscript assessed the applicability and validity of RCTs involving the comparison between minimally invasive and open esophageal surgery by using PRECIS-2 and Risk-of-Bias 2 tools. The results showed that only one out of nine trials was able to be considered as “pragmatic”. Therefore, the authors recommended that the intended purpose of a RCT (explanatory or pragmatic) should be clear when designing the trial.

Overall, the objectives of this review were well defined, the assessment tools and methodologies were properly adopted, the 9 involved RCT reports were thoroughly reviewed and analyzed, the

results were meaningful, and therefore, the conclusion from this review was unbiased, validated and clinically applicable. The reviewer still has a few minor questions for the authors:

1) Regarding the assessment of pragmatism with PRECIS-2 tool, each domain was scored from 1 to 5, were there any “objective” standardized criteria to define a score of 2, 3 or 4 for there specific trials? Can the authors disclose which domains were not agreed initially by the two researchers, but why did one researcher accept the other’s assessment later? Was a thoracic surgeon specialist invited to the discussion when the two independent researchers did not reach agreement on scores initially?

Thank you for your comments. The guidance that accompanies PRECIS-2 describes the 2, 3 and 4 categories and how to score them. This was initially read and then applied to open oesophagectomy and MIO. Each domain was discussed with Prof Blazeby (oesophageal cancer surgeon) to ascertain what normal clinical practice looks like and then discuss what would reduce the score from a 5 (pragmatic). For example, for ‘eligibility’ this might be a reduced age range, or the exclusion of a subset of patients and for ‘follow-up’ this might be more frequent follow-up appointments or tests that aren’t usually run. If there are numerous restrictions e.g., reduced age range AND exclusion of a subset of patients, this would be reduced from 5 by two points to 3. Looking back at our initial assessments, it was the ‘eligibility’, ‘setting’ and ‘primary outcome’ domains that were the most often scored differently between assessors. One assessor was more stringent on certain exclusion criteria, on single centre studies and how patient-orientated pulmonary complications was. The two assessors met to discuss scores and a score was finalised. These were all then discussed with Prof Blazeby and our scoring criteria was refined.

2) The reviewer agree that the trial needs to be pragmatic if aiming to change practice, or need to be explanatory if establishing mechanisms. However, can the authors discuss more on which conclusion should be more reliable if paradoxical conclusions were drawn from a pragmatic trial and an explanatory trial for the same clinical hypothesis, and why?

Thank you for raising this interesting point. It would be important to consider the implications for practice if paradoxical conclusions are drawn from a pragmatic and explanatory trial with the same clinical hypothesis. Whilst this may seem plausible, we consider that this should not happen if a trial has been carefully designed to address a specific detailed clinical hypothesis. A clinical hypothesis would be based on the PICO (patient population, intervention, comparator and outcome). Provided sufficient detail is given for each component, the degree of pragmatism or otherwise can be determined. We have updated the discussion to describe how in trial design it is important to provide

specific details about the clinical hypothesis that inform the degree of pragmatism or otherwise (pg 12, lines 15-16).

Surgery is complex and patients are complex. An explanatory trial may look to control for some of these complexities by excluding the less well people or involve only the most highly-skilled, experienced surgeons which together may show the intervention to be highly successful, but when performed in a normal hospital in the usual patient population, this intervention may not be so successful. We believe that the pragmatic approach, which attempts to reflect usual clinical practice in the usual patient population, is therefore more reliable, since it does not try to control critical elements of the procedural pathway.

Reviewer 4

David Chambers, National Cancer Institute

Comments to the Author:

This is a well-written, exceptionally clear paper that furthers along the conversation about how best to apply available tools to assess the pragmatic vs. explanatory nature of trials. It breaks new ground by applying these tools to surgical interventions, through a review of a specific type of trial, and offers important commentary on the challenges of using the tool, the implication of findings, and potential next steps for the field to consider. The background, methodology, results and discussion are all quite succinct and accessible to the average reader, and the points raised in the review underscore the important issues of applying tools created to assist in design of trials to the summation of trials in a given field.

The article acknowledges limitations inherent in the study, particularly related to the limited information provided in publications to determine appropriate ratings, the particularities of surgical interventions and how they should be interpreted according to specific domains, and the lack of existing guidance on defining a line between exploratory and pragmatic. All of this is handled by the authors extremely well.

There is one particular area that I would suggest would be useful to expand in the discussion. Critical assessment in each domain of the impact of a pragmatic vs. explanatory score might lead to an ability to weight the different domains in terms of deriving a summary score versus the mean. I would appreciate the authors' insights on whether certain domains are naturally less relevant for specific types of studies or interventions, and they would suggest rating a subset of domains or more

heavily weighing some versus others. Implications of a pragmatic score in a domain that may run counter to the type of intervention would be useful for the field to learn and could be done given the team's analysis and expertise. While the authors suggest that better guidance on scoring would be useful, I would appreciate understanding their guidance, given the experience of completing this study. The use of the Risk-of-Bias 2 tool is additive as well, although the study might benefit from more integration (including in the title) of how the PRECIS-2 and ROB-2 tools complement one another. Right now, it seems like the risk of bias aspects of the study are disconnected from the overall discussion.

With consideration of these points, I see the resulting article as one of benefit to the trials community and look forward to seeing a revised discussion should it be needed.

Response:

Thank you for the supportive comments. Over the last decade, numerous checklists, guidelines and tools have been developed to try and improve the design, implementation and reporting of RCTs. However, most of these are less relevant to complex surgical procedures and cannot be applied comprehensively. The PRECIS-2 framework acknowledges its limitations for use in surgical trials with the suggestion that the 'flexibility: adherence' domain is not applicable; however, we have adapted this to reflect surgeons' adherence which is an important aspect of surgical quality assurance.

We have learnt a lot carrying out these assessments for this review and others that we have completed[1]. We found that the PRECIS-2 framework can be applied successfully to surgical interventions following detailed discussions with experts about usual clinical practice in the UK (these judgments are based on our understanding of usual clinical practice in the NHS). During these discussions and preliminary assessments, it became apparent that some domains require more in-depth grading (not binary) than others and require a thorough understanding of the interventions and their clinical application to make an informed judgment. Whilst all domains play a role in assessing pragmatism, we have identified five domains that we believe play a more central role in defining whether a trial has a more explanatory or pragmatic approach. 'Eligibility', 'follow-up' and 'primary outcome' are relevant to all interventions, whereas 'organisation' and 'flexibility: delivery' are especially important when assessing surgical trials.

1. *Eligibility - there are numerous generic (e.g., ASA grade) and/or disease specific (e.g., Siewert/tumour grade) criteria used to select patients for surgical interventions and understanding the relevance of patient exclusions is key to an accurate judgment.*

2. *Follow-up - an understanding of the frequency of follow-ups after an intervention and what assessments are usually done at these appointments e.g., blood tests, scans, quality of life questionnaires, is key.*
3. *Primary outcome – surgeon-orientated outcomes suggest that the study is more focussed on improving the performance of the intervention rather than how it can improve patient outcomes.*
4. *Organisation – looks at whether additional expertise and resources are required to deliver the intervention and considers minimal surgical experience, needs for additional surgical training, and additional equipment. The lack of applicability of the TIME trial to normal clinical practice demonstrates the importance of considering surgeon's experience and training (learning curve).*
5. *Flexibility: delivery – surgical interventions are complex with numerous components and co-interventions. Assessing the reporting of the interventions highlights those studies that lack reporting and those that provide detailed protocols. For surgical trials, the ultimate pragmatic trial would be leaving the surgeons to perform the intervention how they see fit, however, if a study is to be reproducible, then there needs to be a level of detail about how the intervention was performed.*

Analysing just a sub-set would omit the other domains that are still important, whereas weighting the more relevant domains is possible and something we would need to investigate. Given that these assessments require such a considerable amount of work, summarising this as a wheel or a mean value loses a lot of important trial details. Perhaps providing more data alongside a score may improve the value of these assessments to readers, but this requires more thought and perhaps the development of a surgery-specific version of this framework.

In our manuscript, we commented that clearer guidance on the cut-off values that define a trial as pragmatic was needed. The tool was not developed to generate a summary, mean or median score, hence the absence of any guidance on defining a pragmatic or explanatory trial. To understand how best to report the outputs from the PRECIS-2 assessments, we looked at the published literature for studies that had used the PRECIS-2 tool to assess completed studies and found that there were no agreed thresholds. We highlighted this as a weakness in the original manuscript (pg13).

Thank you for highlighting that the ROB assessments appear disconnected. The novelty of this systematic review is the assessment of applicability and hence became the focus of the review.

Reviewer: 5

Dr. Kevin Thorpe

Comments to the Author:

The paper is generally well written. However, I believe if proceeds from a false premise; that pragmatic trials are preferred over explanatory. Therefore, my comments will focus more on context.

First, there appears to be a desire to have cut-offs to declare a trial as pragmatic. This, in fact, is completely contrary to why PRECIS and PRECIS-2 were designed. Due to the multidimensional factors it seemed overly simplistic to reduce trials to a binary classification. The domains actually matter. The push for a classification implies a belief that trials of more pragmatic character are preferred over ones tending to explanatory. I disagree with this.

In this paper, we have tried to emphasise that both explanatory and pragmatic characteristics are acceptable but are dependent on the purpose of the trial. Trials that purport to being applicable to usual practice should be predominantly, if not fully pragmatic. However, we have discussed that in the setting of an RCT, where some aspects of trial design are more explanatory (such as intervention standardisation and monitoring adherence) to enable results to be compared across study centres.

We acknowledge that the way in which PRECIS-2 is applied to published trials is very different from its intended use and is likely the reason for any discordance. We agree that the overall classification of trials as mainly explanatory, equally explanatory/pragmatic and mainly pragmatic is simplistic and certainly does not reflect the amount of work that is required to make these judgments. This is similar to the assessments of risk of bias which also requires analysing information and making decisions, which is condensed to three possible outcomes by the provided algorithm. Whilst the consolidating of information may not be the most ideal method (since so much detail is lost) it does provide the reader with data in a digestible format. In this review, and others we have completed[1], we have provided the rationale behind our decisions for risk of bias assessments, in order to be transparent. When reporting the results from our PRECIS-2 assessments, we have provided some domain-specific information to support our decisions, but perhaps more comprehensive information is required. We have added to the discussion to suggest that readers should look at the individual domain scores on PRECIS wheels to gain a greater understanding of each trial's design (page 14)

We also agree that the domains do matter and in carrying out these assessments over several years, it has become apparent to us that some domains are more relevant to surgical trials (the focus of our work) than others and affect the pragmatism of trials differently. In the discussion of the original manuscript, we have considered the importance of certain domains (pg 11, line 15 to pg 12 1-3).

The difficulties in knowing how to present PRECIS-2 outputs in systematic reviews arise from the use of PRECIS-2 in a format for which it was not designed. We believe the tool is extremely helpful for encouraging the readers to consider many aspects of trial design and has a place in the retrospective assessment of applicability. Going forward, perhaps it could be recommended that when using PRECIS-2 retrospectively, evidence used to support the judgments should be provided. This will ensure that the readers have all the information they require to make an informed decision. This is especially important because the assessment of domains is subjective and are based on what the user deems 'usual clinical practice'.

Second, there is an important characteristic of PRECIS-2 that I believe is frequently overlooked. When PRECIS-2 was developed, the lead authors approached it from the standpoint of developing the tool to help design pragmatic trials. This can be seen from the absence of domains related to the comparator group. In PRECIS-2 the comparator group is assumed to be usual care. The paper does indicate that if your comparator is not usual care you should also perform the PRECIS-2 assessment on it. This becomes very important in light of the first sentence of the discussion which states, "... comparing open versus minimally invasive oesophagectomy to usual clinical practice." The final words of this sentence imply there is also a usual practice that is not reflective of either of the treatment groups considered in the trials reviewed. If my interpretation is correct and the trials reviewed were claimed to be pragmatic by their authors, the situation may be even more grim than the authors of the present paper report.
Thank you for highlighting this interesting nuance of the tool, which we will take forward for future assessments. All the studies in this review compared open oesophagectomy (usual care) to a form of MIO (total or hybrid) (intervention under evaluation). The first line of our discussion is misleading due to the lack of commas. We have added these and the sentence now reads "The review provides an in-depth examination of the applicability of surgical RCTs, comparing open versus minimally invasive oesophagectomy, to usual clinical practice." (page 11, lines 1-2)

One minor point worth raising is that of internal validity, as mentioned by the authors. It is important to keep in mind that internal validity considerations for an explanatory leaning trial should not be applied to the pragmatic leaning trial since the goals are different.
We agree with the reviewer about this comment and have not edited the manuscript any further.

The tendency I have seen in trials is to call them pragmatic in those cases where the investigator

wishes deliver the interventions as they would in practice, However, without careful consideration of all design elements, the generalizability hoped for is likely to suffer. I think the authors have done a good job at showing the apparent lack of attention to the other domains.

In summary, I think this paper could be greatly strengthened by considering what kinds of trials are needed in this field and how the trials reviewed address or fail to address those needs. This is in addition to addressing the two main points above.

We have added to the discussion some views about what kind of trials are needed in this field. We have also added, in addition to addressing the two main points above, our views of where the included trials did not address these needs (i.e. that the trial needs to be explicit in its intention to be pragmatic or explanatory) (page 14).

I hope the authors find my comments helpful and thought-provoking.
We thank you for these extremely helpful and thought-provoking comments – we really appreciate the time taken to give such insightful thoughts and we hope we have done them justice in our responses.

References

1. Chalmers KA, Cousins SE, Blazeby JM, the By-Band-Sleeve Trial Management G. Randomized controlled trials comparing gastric bypass, gastric band, and sleeve gastrectomy: A systematic review examining validity and applicability to wider clinical practice. *Obes Rev* 2024;**25**(5): e13718.

VERSION 2 - REVIEW

**Reviewer**	**3**
**Name**	**Zhang, Peng**
**Affiliation**	**Capital Medical University Affiliated Beijing Friendship Hospital**
**Date**	**07-Aug-2024**
**COI**	**None**

THE COMMENTS AND QUESTIONS HAVE BEEN ADDRESSED

**Reviewer**	**5**
**Name**	**Thorpe, Kevin**
**Affiliation**	
**Date**	**06-Aug-2024**
**COI**	**None.**

Thank-you for the comprehensive reply to my previous review. I only have a couple comments.

On page 10 (page 11 of 63 in the consolidated pdf) you comment on the use of ITT versus per-protocol analyses. As you know, in general the ITT is the approach associated with pragmatic approaches. There is an exception -- non-inferiority (or equivalence) trials. If the trial using per-protocol was a non-inferiority trial, there is no real issue. If any of the ITT analyses were for non-inferiority trials, there is a an issue. As an aside, my personal feeling is that non-inferior trials are less comparable with the pragmatic trial goals, but that is likely beyond the scope of the paper.

I draw your attention to the first sentence of the discussion. You did explain this in your response but I still find the sentence confusing.

VERSION 2 - AUTHOR RESPONSE

Reviewer: 5

Dr. Kevin Thorpe

Comments to the Author:

Thank-you for the comprehensive reply to my previous review. I only have a couple comments.

On page 10 (page 11 of 63 in the consolidated pdf) you comment on the use of ITT versus per-protocol analyses. As you know, in general the ITT is the approach associated with pragmatic approaches. There is an exception -- non-inferiority (or equivalence) trials. If the trial using per-protocol was a non-inferiority trial, there is no real issue. If any of the ITT analyses were for non-inferiority trials, there is a an issue. As an aside, my personal feeling is that non-inferior trials are less comparable with the pragmatic trial goals, but that is likely beyond the scope of the paper.

Response:

Thank you for this important reminder. Of the four trials that provided information about the analyses plans (either reported in paper or from personal correspondence), only one (van der Sluis) specified that it was a superiority trial and the paper states they used ITT analyses. The other three trials did not specify the hypotheses. However, looking at the reasoning for the trial along with the aim and sample size calculations provided, one could surmise that all were looking to test the superiority of the minimally invasive oesophagectomy over the open oesophagectomy for a specific primary outcome of interest. This includes the one trial that used per protocol analysis (Paireder) and was marked down in PRECIS-2 for not using an ITT approach.

We have added this text to the discussion (page 13, lines 18-21) to clarity these important points.

The use of per-protocol analyses in surgical trials is therefore important and often essential in non-inferiority designs. The definitions of a per-protocol analyses in a surgical trial, however, are often confusing and trialists need to carefully describe this in relevant documents to optimise transparency.
I draw your attention to the first sentence of the discussion. You did explain this in your response but I still find the sentence confusing.

Response:

We are sorry that this sentence is still confusing. We have added to the text which we hope will clarify what we mean (page 11, lines 5-10).
